# Negative Pressure Wound Therapy for Closed Surgical Wounds in Musculoskeletal Oncology Patients - A Case-Control Trial

**DOI:** 10.2174/1874325001711010502

**Published:** 2017-05-31

**Authors:** Roderick Kong, David Shields, Oliver Bailey, Sanjay Gupta, Ashish Mahendra

**Affiliations:** Department of Musculoskeletal Oncology, Glasgow Royal Infirmary, 84 Castle Street, Glasgow, G4 0ET Scotland, United Kingdom

**Keywords:** Sarcoma, Negative pressure, Wound, Infection, Surgical site infection, Dressings

## Abstract

Following excision of musculoskeletal tumours, patients are at high risk of wound issues such as infection, dehiscence and delayed healing. This is due to a multitude of factors including the invasive nature of the disease, extensive soft tissue dissection, disruption to blood and lymphatic drainage, residual cavity and adjuvant therapies. The use of negative pressure wound therapy (NPWT) has a growing body of evidence on its beneficial effect of wound healing such as promoting cell differentiation, minimising oedema and thermoregulation. Traditionally, these dressings have been used for open or dehisced wounds; however recent research has investigated its role in closed wounds.

**Aim:**

To evaluate the effect of NPWT in patients with closed wounds, either primarily or with flap coverage, in our high risk group. Consecutive patients who had a NPWT dressing applied were selected, and a control group was established by a blinded researcher with matching for tissue diagnosis, surgical site, gender and age. The primary outcome measured was documented for wound complications, with secondary data collected on radiotherapy and wound drainage.

**Results:**

Patients were well matched between the intervention (n=9) and control (n=9) groups for gender, age and tissue diagnosis. Both groups had 1 patient who underwent preoperative radiotherapy. A total of 3 wound infections occurred in the control group and none in the NPWT group. Overall there was a trend towards lower drain output and statistically significantly reduced infection rate in the NPWT group.

**Conclusion:**

In this short series, despite the NPWT patients having more additional risk factors for wound issues, they resulted in fewer infections. The sample size is not sufficient to have statistically significant reduction. Further evaluation on the value of NPWT in this patient group should be prospectively evaluated.

## INTRODUCTION

The definitive treatment of any sarcoma is resection of the malignant tissue with a margin that is clear of any pathology [1, 2]. Following resection of sarcomas patients are often at high risk of developing complications of surgery such as infection, wound dehiscence and delayed wound healing [3, 4]. The reasons for postoperative wound complications within the cohort of sarcoma patients tend to be multifactorial, although it can be divided into factors due to the nature of the disease and factors resulting from treatment which is initiated. Sarcomas often divide rapidly and have a highly invasive potential. This surrounding poor quality tissue bed is then further compromised with the surgical insult. This can all be worsened by damage to surrounding lymphatic and vascular systems which predispose the area to oedema post operatively resulting in wound complications. Extensive soft tissue dissection with residual cavity also results in oedema which can result in wound issues. Furthermore, with excision of the sarcoma, patients usually require adjuvant radiotherapy or chemotherapy, which are both highly cytotoxic and can delay wound healing [5-7].

The development of negative pressure wound therapy (NPWT) over the last 15 years has been of great value in wound healing. NPWT consists of open-pore foam to fill the cavity, a semi-occlusive dressing with a suction tube connected to a suction device to create a negative pressure environment [8]. The device most commonly used in our unit is the Vacuum Assisted Closure (ActiV.A.C, KCI San Antonio, Texas). Application of the NPWT device helps to promote wound healing on the macro and microscopic level. On the macroscopic level, the use of NPWT dressing induces wound shrinkage caused by the collapse of the pores of the foam or mesh by 80% and centripetal forces are transferred to the wound surface by the dressing [9, 10]. Furthermore, the creation of an environment in subatmospheric pressure with the vacuum device helps greatly in removing excessive fluid from the wound, which is often the case in postoperative sarcoma patients, and can be the contravening factor to wound healing as it can exert compressive forces on surrounding tissue [11]. Microscopically, NPWT helps to induce undulation of the wound surface - called microdeformations- which transmits shear and hydrostatic forces via the extracellular fluid and matrix to the individual cells [12]. The combination of these forces with the pull of gravity results in alteration in the cell shape which promotes cell proliferation, migration and differentiation [13]. These processes are vital in helping with formation of granulation tissue. Furthermore, the microenvironment created by the NPWT activates the neurocutaneous system stimulating neural growth [14] and stimulating the production of neuropeptides which play a role in wound healing. In creating this microenvironment using the foam and semi-occlusive dressing, it creates thermoregulation of the wound. There have also been suggestions that the application of NPWT can help to reduce the bacterial load which can reduce the risk of wound infection but there is no evidence currently to support this.

NPWT has been used in trauma and orthopaedics and allied surgical specialities. Traditionally, NPWT was developed for use on open or dehisced wounds but recent research has shown an increasing use for NPWT in closed wounds. For example, the use of NPWT has been shown to be significant with time for wound healing in drainage of haematomas being 3.1 days without NPWT to 1.6 days for the wound to dry [15]. It has also been utilized in high risk lower extremity fractures, significantly reducing the time for the wound to dry from 4.8 days to 1.8 days [15]. A larger randomized control trial looking at wound complications in high risk lower extremity fracture showed lower rates of deep infection (19% *vs.* 10%) and dehiscence (16.5% *vs.* 8%) [16]. In the elective setting, NPWT has also been applied to primary hip arthroplasties demonstrating a mean reduction in seroma volume from 5.08ml vs 1.97 ml [17]. Outside of orthopaedics, NPWT has also been used in cardiothoracic surgeries for sternotomy wounds in plastic, vascular and abdominal surgery. These have resulted in reduction of wound complications postoperatively [18-21].

## AIM

Our literature search showed little evidence regarding the use of NPWT in postoperative sarcoma patients. The aim of this study was to evaluate the wound healing outcomes in patients with high risk wounds after sarcoma resection treated with and without use of NPWT.

## METHOD

A case controlled study was conducted in patients with closed surgical wounds (either primarily or flap covered) in which the cohorts were divided into an intervention group which was a consecutive series of 9 patients who had NPWT routinely applied for primarily closed sarcomas. This group was matched with a control group from our sarcoma database who had no NPWT. Patients were matched in terms of age, gender, site of the tumour as well as method of closure. The control group was generated at random by an independent investigator from a prospectively collected database. In this study, the primary outcomes used to evaluate wound complications were documented in terms of wound complications in the inpatient notes, additional surgical procedures in the same admission relating to wound breakdown, use of other non-routine antibiotics indicating infection of the wound and drain volume (drains are routinely used postoperatively). We also looked at the use of adjuvant chemotherapy and radiotherapy whether pre or post-surgery in order to take into account confounding factors which may promote wound breakdown. All patients received 40mg of enoxaparin subcutaneously after the operation until discharge as routine prophylaxis against venous thromboembolism.

The NPWT dressing was applied directly to the incision with a mesh and occlusive dressing and attached to a suction device. The dressing was applied for a minimum of 4 days and the decision to remove the dressing was based on the wound review.

To evaluate the non-inferiority of the NPWT group’s statistical difference,a t-test was performed for comparison of means and Z-testing for comparison of proportions using SPSS v23 (SPSS Inc, Chicago, Illinois).

## RESULTS

There were 9 patients from the period of October 2014 to August 2015 who had VAC dressing applied as a routine measure immediately post op. The outcomes of these patients when compared to the matched control group are shown in the tables below, with patient demographics being demonstrated in Fig. (**1**) and the distribution factors which can influence wound healing in Fig. (**2**).

The mean ages for both the NPWT and control group were 56 and 57, respectively. Among these patients, 3 had chemotherapy from the NPWT group and 1 in the control group. Both groups had 1 patient who underwent preoperative radiotherapy. Both the NPWT and control group were also matched for site of the disease with 1 upper limb, 7 lower limb and 1 truncal in each group. In the NPWT group, none of the patients developed wound infection in comparison to 3 patients in the controlled group. One patient in the NPWT group developed flap necrosis which had to be treated with prolonged VAC dressing.

VAC dressings in the NPWT group were applied for a mean of 5.4 days. The control group showed a higher volume from the wound drains (525ml versus 338ml in the NPWT group). Although there was a trend to lower volume drains, there were insufficient numbers to show a statistical difference (p=0.335). Comparison of proportion of wound issues was statistically significant (p=0.0287).

## DISCUSSION

The use of NPWT as a routine measure in sarcoma patients has the potential to improve the quantitative and qualitative aspects of patient outcome. In terms of quantitative measures, wound complications can lead to increased healthcare costs in terms of increased antibiotic use and prolonged hospital stay. Further surgical interventions such as irrigation and debridement or wound revision lead to increased cost. Qualitatively, prolonged hospital stay due to wound complications results in prolonged immobilisation which can limit the quality of life and functional outcomes of patients postoperatively. Delayed mobilization can result in deconditioning and compromised function. These factors can also impact patient satisfaction and psychological recovery.

The cost of a reusable ActiVAC unit in our institution is £6,500 with the individual dressings costing approximately £25 each. Given the price, the use of NPWT as routine measure may incur an increase in the treatment cost. However, the efficacy of NPWT has been shown by the results of this study and this can have the potential to reduce overall hospital cost by reducing the length inpatient stay, along with reducing need for further interventions (antibiotics, surgery, additional dressings *etc*.). Current estimates in the UK for daily bed cost vary between regions, however an estimation of £400 per bed per day is often quoted by the Department of Health. Our institution manages approximately 50 sarcomas per year. If NPWT were to save 0.35 bed days per patient, the cost savings would offset the expense of the use of the portable NPWT machine. By improving the wound care, we can also improve patient satisfaction and their functional outcome. The direction of bias in this study favoured the NPWT group (more post-operative chemotherapy, higher mean age, more specialist plastic surgery input) and despite this, the NPWT group had fewer complications. In order to build on this level 4 evidence, a prospective study into this has been initiated in our unit to elaborate further on the effect of NPWT on primarily closed sarcoma wounds.

There is increasing evidence regarding the benefit of NPWT in primarily closed wounds. The use of NPWT in trauma is well established in demonstrating a decrease in wound complications [15, 16, 22-24], with improvements seen in terms of reducing time for the wound to dry, percentage of dehiscence and signs of deep infection. Although primary outcome varied between the studies, the ultimate improvement in patient care seen in this trial included wound infection & breakdown rates. Any future analysis could prospectively monitor surgical site infection rates, and perform cost-analysis to justify the initial investment of routine use of NPWT dressings.

## CONCLUSION

Use of NPWT trended towards a clinical improvement in primarily closed and flap covered surgical wounds after sarcoma resection could improve not just the quality of life and the patient care, but also may give rise to financial savings in the long term. An appropriately powered prospectively randomised controlled trial within a cohort of sarcoma patients is ethically approved and currently recruiting within our institution and aims to expand to multiple centres in the near future (clinicaltrials.gov - NCT02901405).

## Figures and Tables

**Fig. (1):**
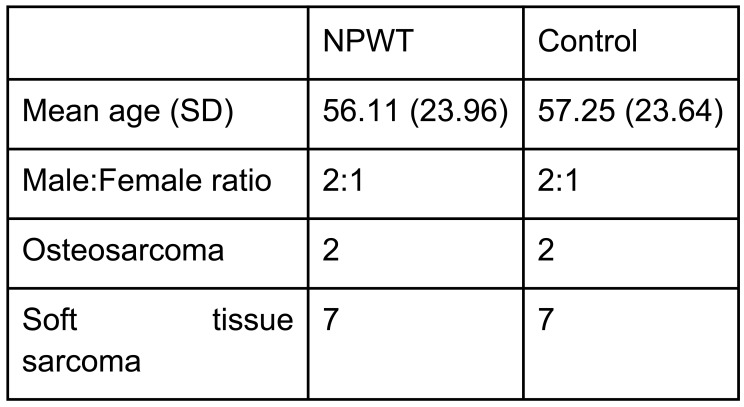
Table showing the demographic of the study group.

**Fig. (2):**
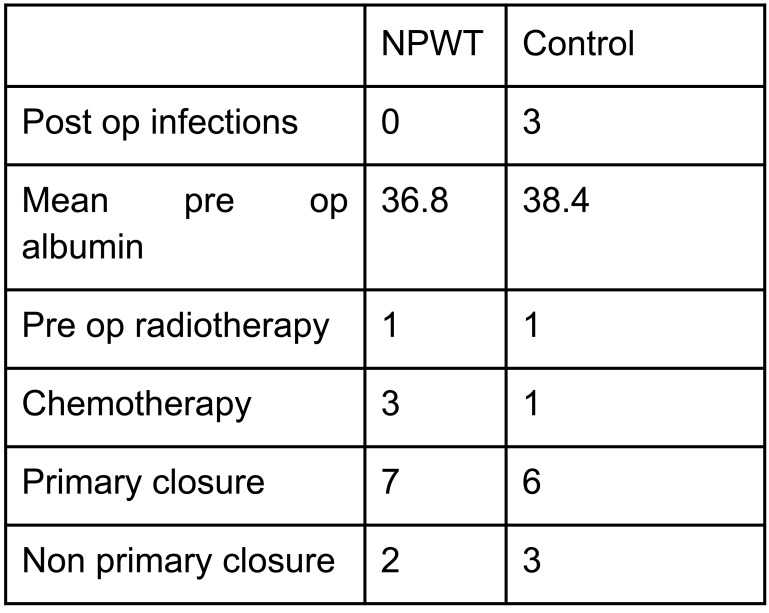
Table showing the distribution of factors which can influence wound healing.

## LIST OF ABBREVIATIONS

NPWT: = Negative pressure wound therapy
VAC: = Vacuum assisted closure
